# Sleep problems increase the risk of musculoskeletal pain in boys but not girls: a prospective cohort study

**DOI:** 10.1007/s00431-020-03667-8

**Published:** 2020-05-12

**Authors:** Alessandro Andreucci, Paul Campbell, Lisa K Mundy, Susan M Sawyer, Silja Kosola, George C Patton, Kate M Dunn

**Affiliations:** 1 Primary Care Centre Versus Arthritis, School of Primary, Community and Social Care, Keele University, Keele, Staffordshire ST5 5BG UK; 2 Center for General Practice at Aalborg University, Department of Clinical Medicine, Aalborg University, 9220 Aalborg Ost, Denmark; 3 Midlands Partnership NHS Foundation Trust, Department of Research and Innovation, St Georges Hospital, Corporation Street, Stafford, Staffordshire ST16 3SR UK; 4 Murdoch Childrens Research Institute, Royal Children’s Hospital Centre for Adolescent Health and Department of Paediatrics, The University of Melbourne, Flemington Rd Parkville, Melbourne, Victoria 3052 Australia; 5 Children’s Hospital, Helsinki University Hospital and University of Helsinki, Helsinki, Finland

**Keywords:** Sleep problems, Musculoskeletal pain, Risk factor, Children, CATS study

## Abstract

Adults with sleep problems are at higher risk for onset of musculoskeletal pain, but the evidence is less clear for children. This prospective cohort study investigated whether children with sleep problems are at higher risk for onset of musculoskeletal pain and explored whether sex is a modifier of this association. In a prospective cohort study of Australian schoolchildren (*n* = 1239, mean age 9 years), the associations between sleep problems at baseline and new onset of both musculoskeletal pain and persistent musculoskeletal pain (pain lasting > 3 months) 1 year later were investigated using logistic regression. The potential modifying effect of sex was also assessed. One-year incidence proportion for musculoskeletal pain onset is 43% and 7% for persistent musculoskeletal pain. Sleep problems were associated with musculoskeletal pain onset and persistent musculoskeletal pain onset in boys, odds ratio 2.80 (95% CI 1.39, 5.62) and OR 3.70 (1.30, 10.54), respectively, but not girls OR 0.58 (0.28, 1.19) and OR 1.43 (0.41, 4.95), respectively.

*Conclusions*: Rates of musculoskeletal pain are high in children. Boys with sleep problems are at greater risk of onset of musculoskeletal pain, but girls do not appear to have higher risk. Consideration of sleep health may help prevent persistent musculoskeletal pain in children.

**Table:** 

**What is Known:** • *Sleep problems are associated with the onset of musculoskeletal pain in adults.* • *It is not clear if the association between sleep problems and the onset of musculoskeletal pain is present also in children and if sex plays a role in this association.*	
**What is New:** • *This is the first large population-based study that has prospectively investigated the relationship between sleep problems and onset of musculoskeletal pain in school-aged children.* • *Children, especially boys with sleep problems, were at increased risk for the development of persistent musculoskeletal pain.*

**What is Known:**

• *Sleep problems are associated with the onset of musculoskeletal pain in adults.*

• *It is not clear if the association between sleep problems and the onset of musculoskeletal pain is present also in children and if sex plays a role in this association.*

**What is New:**

• *This is the first large population-based study that has prospectively investigated the relationship between sleep problems and onset of musculoskeletal pain in school-aged children.*

• *Children, especially boys with sleep problems, were at increased risk for the development of persistent musculoskeletal pain.*

## Introduction

Musculoskeletal pain is a major concern worldwide, with conditions such as low back pain and neck pain ranking highly in years lived with disability (YLDs) in those aged 10 to 19 years old [1]. Up to 40% of children experience musculoskeletal pain [2], which can become persistent and impact daily living (e.g. social, educational and physical activities), result in disability, increase healthcare use and precipitate regular use of analgesia [3, 4]. Musculoskeletal pain in adults may have its origin in childhood, highlighting the need to understand risk factors within child and adolescent populations [5].

One potential risk factor is the presence of sleep problems, with prospective studies in adults suggesting a link between poor sleep and the onset of musculoskeletal pain [6]. In children, sleep problems (e.g. bedtime problems, night waking, sleep related anxiety, deficient sleep and poor sleep hygiene) are common, with up to 40% of children experiencing at least one type of sleep problem during childhood [7–9]. Previous reviews have shown clear cross-sectional associations between pain and sleep problems in children [10]. However, prospective evidence is mixed with effects dependent on certain sub-groups such as age, body area and sex, suggesting potential effect modification [11]. Regarding sex, insufficient sleep quantity or quality was a risk factor for low back pain and neck pain in girls in one study [12]; however, in another study, insufficient sleep was a risk factor for musculoskeletal pain only in boys [13]. Other factors may also affect the relationship between sleep and musculoskeletal pain, including levels of physical activity [14, 15] and the presence of psychological symptoms [8, 16–18]. For example, children with psychological symptoms such as attention deficit hyperactivity disorder (ADHD) might also experience specific types of sleep problems such as the restless legs syndrome [19]. Restless legs syndrome can occur together with growing pain or without, in which case children with restless legs syndrome might still report symptoms that are misdiagnosed for growing pains [20], thus showing the complexity of the relationship between sleep and pain.

The aim of this study was to prospectively test whether children aged 8–9 years old with sleep problems are at higher risk for the onset of musculoskeletal pain and persistent musculoskeletal pain 1 year later, compared to those without sleep problems. The second aim was to investigate whether sex is an effect modifier of this relationship.

## Methods

### Design

The study was a secondary data analysis of a longitudinal prospective cohort study.

### Participants

**Fig. 1:**
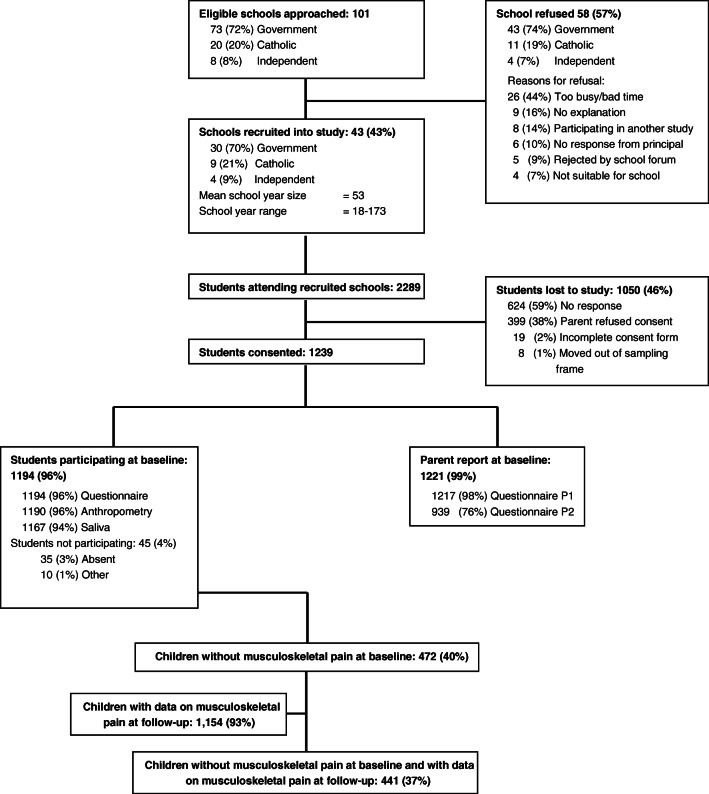
Flowchart of participants from baseline to follow-up

The study population were participants in the Childhood to Adolescence Transition Study (CATS). CATS is a longitudinal study of children assessing several health and learning outcomes during puberty [21]. The cohort includes information gathered from both schoolchildren and their parents within metropolitan Melbourne, Australia. Primary schools with 10 or more children enrolled in grade 3 were randomly selected from a stratified cluster sample (government, catholic, independent schools). Parents provided consent for parent and child participation. Recruited children were 8–9 years old (grade 3) at baseline and were followed-up 1 year later. Figure 1 shows the process of recruitment for both children and parents. Further information about the study can be found in the study protocol [21].

### Measures

#### Pain

Musculoskeletal pain was assessed at baseline and follow-up using a self-report question that has been previously used in child cohort studies [16, 17]: “Thinking back over the PAST MONTH, have you had any pain or pains, which have lasted for a WHOLE DAY or LONGER?”. If the response was “yes”, children were asked if their pain started more than 3 months before the assessment or not, as an indicator of persistent pain. A pain manikin was used to assess a total of 17 different pain sites in the front and back of the body: head, neck/throat, thoracic spine, upper back, lumbar spine, lower back, chest, abdomen, shoulder, elbow, forearm, hand, buttock, thigh, knee, shin/calf and foot. Such measures have been shown to be reliable in children from the age of 8 years [22, 23]. The answer to the pain question (yes/no) together with the body sites indicated in the pain manikin (excluding those relative to the head and abdomen) were used to classify participants as “having musculoskeletal pain” or “not having musculoskeletal pain”, respectively, at each time point. Incident cases (new onset of musculoskeletal pain) were those who reported not having musculoskeletal pain at baseline and reported having musculoskeletal pain at follow-up. Incident cases for persistent musculoskeletal pain were those who reported not having musculoskeletal pain at baseline but then reported musculoskeletal pain at follow-up that had persisted for 3 months or more at the time of assessment.

#### Sleep problems

Sleep problems were assessed at baseline through a single self-report question for each child: “How often have you been bothered by trouble sleeping in the last month?” (Never/Almost never/Sometimes/Often/Almost always). Responses were dichotomized into two groups: “No sleep problems” (Never/Almost never/Sometimes) and “Sleep problems” (Often/Almost always) as used in previous research in adults [24].

#### Potential effect modifiers and confounders

Sex was analysed as a potential effect modifier.

Psychological symptoms, which may affect both sleep and pain perception, were included as a potential confounder [8, 16–18]. Psychological symptoms were measured (by parent report at baseline) through the total difficulties scale of the Strengths and Difficulties Questionnaire (SDQ). The SDQ produces a continuous score for psychological symptoms, which is valid and suitable for the assessment of behavioural and emotional disorders for children [21, 25]. Also, physical activity may be associated both with sleep problems and musculoskeletal pain [14, 15]. Physical activity was assessed at baseline through a parent-reported question adapted from the Longitudinal Study of Australian Children, “In the last 12 months has your child regularly participated in any of the following activities (outside school hours, even if organised by the school)? (Team sport/Individual sport)”. Possible answers were “yes” or “no” [21].

#### Data analysis

Baseline descriptive data are shown as means and standard deviations (SD) or as counts (%) where appropriate. The *t* test or Pearson *χ*^2^ test was used to compare groups of continuous and categorical variables, respectively. To maximize statistical power and increase precision (i.e. to limit the possibility of a biased estimate) within the logistic regression analysis, multiple imputation with chained equations was applied to impute missing data. In order to minimize bias in the analysis model, all the variables used in the analysis were included in the imputation model [26, 27]. The outcome was also included in the imputation model but not imputed. Following guidance, we created a number of datasets higher than the highest percentage of missing data among the variables [27]. A Little’s test of missing completely at random (MCAR) was performed prior to imputation. Based on the result of Little’s test, data were assumed to be MCAR and therefore imputed. The association between sleep problems at baseline and onset of new musculoskeletal pain at follow-up was assessed by logistic regression with 95% confidence intervals (CI). The logistic regression analysis was repeated to assess the odds of persistent musculoskeletal pain at follow-up. Associations were adjusted for psychological symptoms (SDQ score) and participation in individual and team sport. Analyses were stratified to assess whether sex is a potential effect modifier, with comparisons made between stratified groups, and statistically tested by means of interaction terms. All statistical analyses were conducted using STATA 14.

## Results

### Recruitment

Overall, 1239 children consented to take part to the study (Fig. 1). A total of 1194 children and 1221 parents provided data at baseline, of which 542 boys and 642 girls provided data on trouble sleeping (*n* = 1184). Of these, 1107 (93%) children participated at follow-up. Inspection of missing data due to loss to follow-up (6.7%) showed no statistically significant difference in the baseline characteristics between children who participated at follow-up and those who did not.

### Baseline characteristics

Table 1 outlines the baseline characteristics of the cohort. Reports of any musculoskeletal pain and persistent pain were common, affecting about 60% and 15% of children (no sex differences; *χ*^2^ test, *P* = 0.29). The incidence of musculoskeletal pain at follow-up was 43%, and the incidence of persistent musculoskeletal pain at follow-up was 7%. Among the 718 children who reported musculoskeletal pain at baseline, 670 were still present at follow-up, of which 458 (68%) reported musculoskeletal pain and 97 (14%) persistent pain. Among the 180 children that reported persistent pain at baseline, 166 were still present at follow-up and 30 (17%) of them reported persistent pain. Just over a third (35%) of children reported sleep problems at baseline with similar proportions for boys and girls (_*x*_^2^ test, *P* = 0.54). Post hoc inspection of the children who reported sleep problems showed that 53% of girls vs. 33% of boys had trouble sleeping “often”, while 47% of girls vs. 67% of boys reported “almost always”.

**Table 1:** Baseline sample characteristics

General characteristics	Boys (*N* = 542)	Girls (*N* = 642)	Overall (*N* = 1184)	Missing (%)
Age (years, mean ± SD)	9.0 ± 0.4	9.0 ± 0.4	9.0 ± 0.4	0
Age (median)	9.0	8.9	9.0	
Sleep problems				
Having sleep problems (often/almost always)	185 (34.1%)	230 (35.8%)	415 (35.1%)	4.4
Musculoskeletal pain				
Yes	339 (62.0%)	379 (58.9%)	718 (60.3%)	4.0
No	208 (38.0%)	264 (41.1%)	472 (39.7%)	4.0
Persistent musculoskeletal pain				
Yes	84 (15.4%)	96 (14.9%)	180 (15.1%)	4.0
No	462 (84.6%)	547 (85.1%)	1009 (84.9%)	4.0
Psychological characteristics				
SDQ score (total difficulties, mean ± SD)	8.9 ± 5.8	7.9 ± 5.1	8.4 ± 5.5	3.0
SDQ score (median)	8.0	7.0	8.0	
Physical activity				
Individual sport (yes)	273 (73.4%)	319 (73.5%)	592 (73.4%)	26
Team sport (yes)	275 (74.3%)	229 (54.3%)	504 (63.6%)	27

*SDQ* Strengths and Difficulties Questionnaire, *SD* standard deviation

### Association between sleep problems at baseline and musculoskeletal pain onset at follow-up

Overall, the odds for the onset of musculoskeletal pain were not significantly increased at follow-up among children with sleep problems (adjusted odds ratio, OR 1.35; 95% CI 0.84, 2.17) (Table 2). However, analysis stratified by sex showed that boys with sleep problems were at higher odds for musculoskeletal pain onset (adjusted OR 2.80; 95% CI 1.39, 5.62), whereas there was no increase in odds among girls (OR 0.58; 95% CI 0.28, 1.19). Interaction testing showed a statistically significant effect modification of sex (OR 3.90; 95% CI 1.48, 10.24), indicating a multiplicative effect of male sex on the risk originating from the presence of sleep problems.

**Table 2:** Logistic regression and 95% confidence intervals (CI) of the association between sleep problems at baseline and musculoskeletal (MSK) pain onset at follow-up

**Unadjusted analysis**			
	**Overall (*N*** **= 441)**	**Boys (*N*** **= 196)**	**Girls (*N*** **= 245)**
**MSK pain onset**	**OR (95% CI)**	**OR (95% CI)**	**OR (95% CI)**
Sleep problems	1.47 (0.93, 2.31)	2.77 (1.39, 5.54)	0.87 (0.46, 1.62)
Adjusted analysis*			
	**Overall (*N*** **= 441)**	**Boys (*N*** **= 196)**	**Girls (*N*** **= 245)**
**MSK pain onset**	**OR (95% CI)**	**OR (95% CI)**	**OR (95% CI)**
Sleep problems	1.35 (0.84, 2.17)	2.80 (1.39, 5.62)	0.58 (0.28, 1.19)
Interaction term*		**Sex # sleep**	
		3.90 (1.48, 10.24)	

Analysis were performed on the datasets resulting from multiple imputation with chained equations

*MSK* musculoskeletal

*Analysis adjusted for psychological symptoms (total difficulties score), individual sports and team sports

### Association between sleep problems at baseline and persistent musculoskeletal pain at follow-up

Table 3 shows the association between sleep problems at baseline and persistent musculoskeletal pain at follow-up. Overall, children who reported sleep problems were at significantly higher odds of developing persistent musculoskeletal pain (adjusted OR 2.48; 95% CI 1.15, 5.37). Stratified analysis showed that boys with sleep problems had higher odds for persistent musculoskeletal pain (adjusted OR 3.70; 95% CI 1.30, 10.54), but there was no significant increase for girls (OR 1.43; 95% CI 0.41, 4.95). Interaction testing of sex was insignificant (OR 2.18; 95% CI 0.46, 10.36).

**Table 3:** Logistic regression and 95% confidence intervals (CI) of the association between sleep problems at baseline and persistent musculoskeletal (MSK) pain at follow-up

**Unadjusted analysis**			
	**Overall (*N*** **= 441)**	**Boys (*N*** **= 196)**	**Girls (*N*** **= 245)**
**Persistent MSK pain**	**OR (95% CI)**	**OR (95% CI)**	**OR (95% CI)**
Sleep problems	2.69 (1.28, 5.67)	3.52 (1.27, 9.76)	1.95 (0.64, 5.99)
Adjusted analysis*			
	**Overall (*N*** **= 441)**	**Boys (*N*** **= 196)**	**Girls (*N*** **= 245)**
**Persistent MSK pain**	**OR (95% CI)**	**OR (95% CI)**	**OR (95% CI)**
Sleep problems	2.48 (1.15, 5.37)	3.70 (1.30, 10.55)	1.43 (0.41, 4.95)
Interaction term*		**Sex # sleep**	
		2.18 (0.46, 10.36)	

Analysis were performed on the datasets resulting from multiple imputation with chained equations

*MSK* musculoskeletal

*Analysis adjusted for psychological symptoms (total difficulties score), individual sports and team sports

## Discussion

In this study, new onset of pain, including persistent pain, was common in 8–9 years old children. The presence of sleep problems led to a significant increase in the odds of persistent musculoskeletal pain and an increase in the odds of musculoskeletal pain onset, but stratification revealed that this effect was only present among boys but not girls.

Previous evidence of a relationship between sleep problems and musculoskeletal pain onset in children is mixed. A recent systematic review [11] reported no overall effect of sleep problems on musculoskeletal pain onset which is in accord with our overall findings. The systematic review argued that mixed findings were not only explained by the heterogeneity of included studies (population mix, different measures) but also showed that sex differences explained variation, suggesting potential effect modification. Our study, by specifically examining sex as a potential effect modifier, offers greater clarity on this issue. Our findings show that boys are consistently at an increased risk for the onset of both musculoskeletal pain (with this result supported by a significant interaction term of a quite strong effect size) and persistent pain, whereas girls are not. Despite the previous mixed results [11], a 2-year prospective study [13] reported that sleep quantity and musculoskeletal pain onset were associated only in boys, consistent with our findings. One reason for the increased risk in boys in comparison to girls may be severity of sleep problems. In our sample, more girls (53%) compared to boys (33%) had trouble sleeping “often”, while more boys (67%) compared to girls (47%) reported trouble sleeping “almost always”, suggestive that increasing levels of sleep problems were associated with higher likelihood of the onset of musculoskeletal pain. There might be also important age/sex interactions where risk and outcome change. Some literature supports this view. A study [28] (*n* = 700) showed a slightly higher level of sleep problems in boys within preadolescent stages (age 5–10 years), though notably, the difference is reversed during adolescence (higher in girls aged 11–12 years). The prevalence of pain (especially chronic) is consistently higher in females from early adulthood to older age [29, 30], and a recent study has shown a lower prevalence of consultations for musculoskeletal conditions in girls compared to boys before age 15, but the opposite after age 15 [31]. In another study, insufficient sleep quantity or quality was a risk factor for neck pain and low back pain onset only among girls; the key difference with our study was that participants were older (17 years old at follow-up) in that study [12].

Several biological mechanisms may explain the association between sleep and musculoskeletal pain, including an increased production of cytokine and inflammatory mediators [12, 32], increased muscle tension in individuals with sleep problems [12, 33] or modification of the opioid/dopamine neurotransmission systems, which may result in reduction of pain thresholds [32–34]. Psychosocial explanations include daytime tiredness and fatigue from poor sleep which relate to higher perception of pain [35] and the relationship of sleep problems with depression, both of which independently relate to pain [32, 36]. While this current study adjusted for the influence of psychological symptoms within the analysis, children are more likely to report depressive symptoms as somatic symptoms than adults, and therefore, “masked” depression may explain the link between sleep problems and pain. Psychological symptoms may also offer some explanation for the stronger risk effect in boys. Examination of the SDQ scores (Table 1) shows higher scores for boys, and prevalence of pain is elevated in children with ADHD, which is predominant in boys [37]. In addition, behaviour may also explain the sex difference reported in this study: although both girls and boys report similar individual sport participation (73%), boys participated in team sport more often than girls (girls = 54%, boys = 74%) and boys are more likely to sustain musculoskeletal injury from sport participation both as individuals and within team sports [38, 39]. Sleep problems may also have a role in promoting the persistence or worsening of musculoskeletal pain [32]. This current study conducted exploratory analysis in a sub-group of children with musculoskeletal pain at baseline, and results showed that sleep problems were significantly associated with the presence of musculoskeletal pain and persistent pain at follow-up in this sub-group (data available from authors on request).

To our knowledge, this is the first study that investigated the association between sleep problems and musculoskeletal pain in schoolchildren aged 8–9 years old. A major strength is the prospective cohort design, which provides incidence estimates and allows an understanding of the temporal sequence between exposure and outcome [40]. Another strength is the specific focus on sex as a potential effect modifier, which highlighted groups within the population that may be at increased risk. In addition, multiple imputation with chained equations was conducted to increase precision of estimates. Complete case analysis was also carried out, and results were very similar for both persistent and musculoskeletal pain onset (data available from authors on request). Some limitations should be noted. This study was a secondary data analysis of a cohort not specifically designed for exploring the effect of sleep problems on musculoskeletal pain, which explains why objective measures of sleep (e.g. polysomnography or actigraphy) and self-report diaries and clinical examination for pain were not undertaken [41, 42]. Furthermore, the reports of musculoskeletal pain may have included acute episodes of pain due to an injury which may be transient; additional information (i.e. pain intensity, frequency and the impact of pain such as pain interference or disability) [43] would have provided a greater understanding of the effects sleep has on musculoskeletal pain and may also have elicited more information on at risk groups of clinical relevance (i.e. high pain and high impact). In addition, a proportion of the musculoskeletal pain reported by children might be growing pain, which might overlap with restless legs syndrome [19]. However, the methods used to assess musculoskeletal pain in this study have been used previously [16, 17, 22] and are considered reliable in children from the age of 8 years [22, 23]. Also, both sleep and musculoskeletal pain have been assessed at single time points, with a 1-month recall period. As both sleep and musculoskeletal pain can fluctuate over time, some cases of sleep problems or musculoskeletal pain may have been missed; this could affect the estimate of association. Trajectory studies with assessment of sleep and musculoskeletal pain at more frequent time points would allow a more precise estimate of the relationship between sleep and musculoskeletal pain. In addition, the analyses were adjusted for psychological factors and physical activity, which have been proposed as potential mediators of the sleep-pain relationship. As a result of this adjustment, the total effect of sleep on pain may have been under-estimated. Finally, the follow-up period was only 1 year, which may be considered a relatively brief period in the study of musculoskeletal pain development. Future studies with a longer prospective duration and more frequent follow-up time points would enable exploration of developmental and reciprocal relationships between sleep problems and musculoskeletal pain.

This study found that sleep problems elevate the risk of persistent musculoskeletal pain in children and that only boys were at greater risk of musculoskeletal pain onset. These results provide a platform to better understand the association between sleep and pain, as well as the direction of the association and indicate a sub-group of children at increased risk. From a public health standpoint, different strategies may be effective in preventing persistent musculoskeletal pain, if future research supports these results. This includes greater monitoring of the sleep health of children (by promoting good sleep hygiene in schools or identifying children with sleep problems using a Pediatric Sleep Toolkit) [44] as well as closer monitoring of musculoskeletal pain onset in children with elevated sleep problems in order to identify those at early risk for whom treatment with sleep therapies (e.g. cognitive behavioural therapy) may be effective rather than relying on medication [45–47].

## Conclusions

This study provides prospective evidence of links between sleep problems and musculoskeletal pain in children, with important new information about an increased risk in boys. These associations may be explained by a combination of biological, psychological and behavioural mechanisms, and more research is now required to understand these relationships.

## Abbreviations

CATS: Childhood to Adolescence Transition Study
OR: Odds ratio
95% CI: 95% Confidence intervals
